# Transcranial magnetic stimulation from healthy brain aging to Alzheimer’s disease: a review on mechanisms, therapeutic potential, and future clinical directions

**DOI:** 10.3389/fnagi.2026.1778992

**Published:** 2026-05-08

**Authors:** Faddi Saleh Velez, Maria Cedeno-Bruzual, Melba Zuniga-Gutierrez, Daniela Mercado Pena, Cade L. Ballard, Jeng S. Kong, Ana Clara Da C. Pinaffi-Langley, Cameron D. Owens, Zsuzsanna TucsekCardon, Zsofia Szarvas, Mihaly Muranyi, Zalan Kaposzta, Peter Mukli, Laura Boada Robayo, Stefano Tarantini, Zoltan Ungvari, Andriy Yabluchanskiy, Camila B. Pinto

**Affiliations:** 1 Brain Stimulation and Neurorehabilitation Laboratory, Department of Neurology, University of Oklahoma Health Campus, Oklahoma City, OK, United States; 2 Doctoral College, Health Sciences Division, Semmelweis University, Budapest, Hungary; 3 Fodor Center for Prevention and Healthy Aging, Semmelweis University, Budapest, Hungary; 4 Brain Stimulation and Neurorehabilitation Laboratory, Department of Neurosurgery, University of Oklahoma Health Campus, Oklahoma City, OK, United States; 5 Vascular Cognitive Impairment, Neurodegeneration and Healthy Brain Aging Program, Department of Neurosurgery, University of Oklahoma Health Sciences, Oklahoma City, OK, United States; 6 Department of Nutritional Sciences, College of Allied Health, University of Oklahoma Health Sciences, Oklahoma City, OK, United States; 7 Institute of Preventive Medicine and Public Health, Semmelweis University, Budapest, Hungary; 8 Institute for Translational Research, Budapest, Hungary; 9 Department of Health Promotion Sciences, College of Public Health, University of Oklahoma Health Sciences, Oklahoma City, OK, United States; 10 Peggy and Charles Stephenson Cancer Center, University of Oklahoma Health Sciences, Oklahoma City, OK, United States

**Keywords:** aging, Alzheimer’s disease, cognitive impairment, MCI, neuroplasticity, transcranial magnetic stimulation

## Abstract

Transcranial magnetic stimulation (TMS), a non-invasive and non-pharmacological intervention, is increasingly being explored for mitigating age-related cognitive decline and dementia. Its therapeutic potential is largely attributed to its capacity to modulate neuronal firing rates and induce neuroplastic changes and modulate neurovascular coupling within distributed neural networks that support memory, attention, and executive function. Despite growing interest, gaps remain in understanding how specific stimulation parameters engage in neural circuits and translate into meaningful cognitive outcomes in aging and neurodegeneration. This narrative review synthesizes current evidence on the neurophysiological and hemodynamic mechanisms and clinical effects of repetitive TMS (rTMS) across the spectrum of cognitive aging, from healthy older adults to those experiencing age-related cognitive decline and Alzheimer’s disease. By integrating mechanistic and clinical perspectives, this review bridges basic neuroscience and clinical practice, highlighting rTMS’s emerging role in promoting cognitive resilience and healthier brain aging.

## 1 Introduction

The aging process is commonly accompanied by cognitive decline, signifying profound changes within the central nervous system’s structure, functionality, and biochemistry (Seidler et al., 2010; Rossini et al., 2015). Recent reports show that about 10% of U.S. adults 65 years of age and older have some type of dementia (Major et al., 2026), and about 22% experience mild cognitive impairment (MCI) (Manly et al., 2022). Despite the major health impact, there are only a few pharmacological interventions approved by the Food and Drug Administration (FDA) for the treatment of cognitive decline; moreover, effects are modest and transient, and some therapies have serious side effects (van Dyck et al., 2023).

In this context, non-invasive brain stimulation approaches, such as transcranial magnetic stimulation (TMS), emerge as compelling, non-pharmacological alternatives (Aksu et al., 2024a,b; Gamage et al., 2025; Hall et al., 2024; Lischke et al., 2024; Wojtecki et al., 2024). Given its efficacy across a range of psychiatric disorders (Lefaucheur et al., 2020; Somani and Kar, 2019), it has been recently recognized as possibly effective (Level C evidence) for enhancing cognitive outcomes in patients with MCI and Alzheimer’s disease (AD) (Lefaucheur et al., 2020). By generating a rapidly changing magnetic field that, ultimately, leads to depolarization of neurons, TMS has minimal side effects, an absence of observed detrimental cognitive impacts, and no drug interactions (Ziemann et al., 2015). These attributes position TMS as an auspicious treatment approach for older individuals at risk of cognitive decline and dementia. The adoption of repetitive TMS (rTMS) as intervention is grounded in its ability to induce lasting changes in neuronal function (Drumond Marra et al., 2015; Lefaucheur et al., 2014; Xia et al., 2017). This is achieved through the modulation of neuronal excitability in targeted brain regions (Ziemann and Siebner, 2015).

Despite increasing research, the clinical application of TMS for improvement of cognitive outcomes in aging remains fragmented. This fragmentation reflects meaningful interindividual differences in baseline physiology, network organization, and disease burden, suggesting that variability is not only a methodological obstacle but a central issue for mechanistic interpretation and clinical translation. Studies differ widely in stimulation protocols, targeted brain regions, and measured outcomes, making it challenging to determine the most effective approaches. The mechanisms by which TMS modulates neural networks to improve cognitive outcomes in older adults and those with neurodegenerative conditions are not yet fully understood. Addressing these gaps is essential to optimize TMS interventions and translate mechanistic insights into practical clinical strategies. This review examines the current evidence on rTMS-induced cognitive changes, integrating findings from neurophysiological, experimental, and clinical studies. By considering healthy older adults, individuals with MCI, and patients with AD, it provides a comprehensive perspective on how TMS can affect cognitive outcomes across the aging spectrum and informs future directions for research and clinical implementation, positioning rTMS as a versatile tool in age-related cognitive care.

## 2 Methods

This narrative review was conducted in accordance with SNARA guidelines through a PubMed search from database inception through October 2025. To build a comprehensive narrative around the evidence, we examined randomized controlled trials, observational studies, clinical guidelines, experimental work, and meta-analyses published in the past 5 years that reported effect sizes for rTMS on general cognition, attention, or memory by comparing active with sham stimulation. These sources were used to interpret mechanisms, clarify conceptual issues, and contextualize the evidence. Insights were integrated from supporting studies to describe methodological strengths, limitations, and areas of disagreement within literature. Throughout, this review explicitly addresses sources of bias and heterogeneity to provide a balanced account of the state of the evidence.

## 3 TMS mechanisms and physiology

**FIGURE 1:**
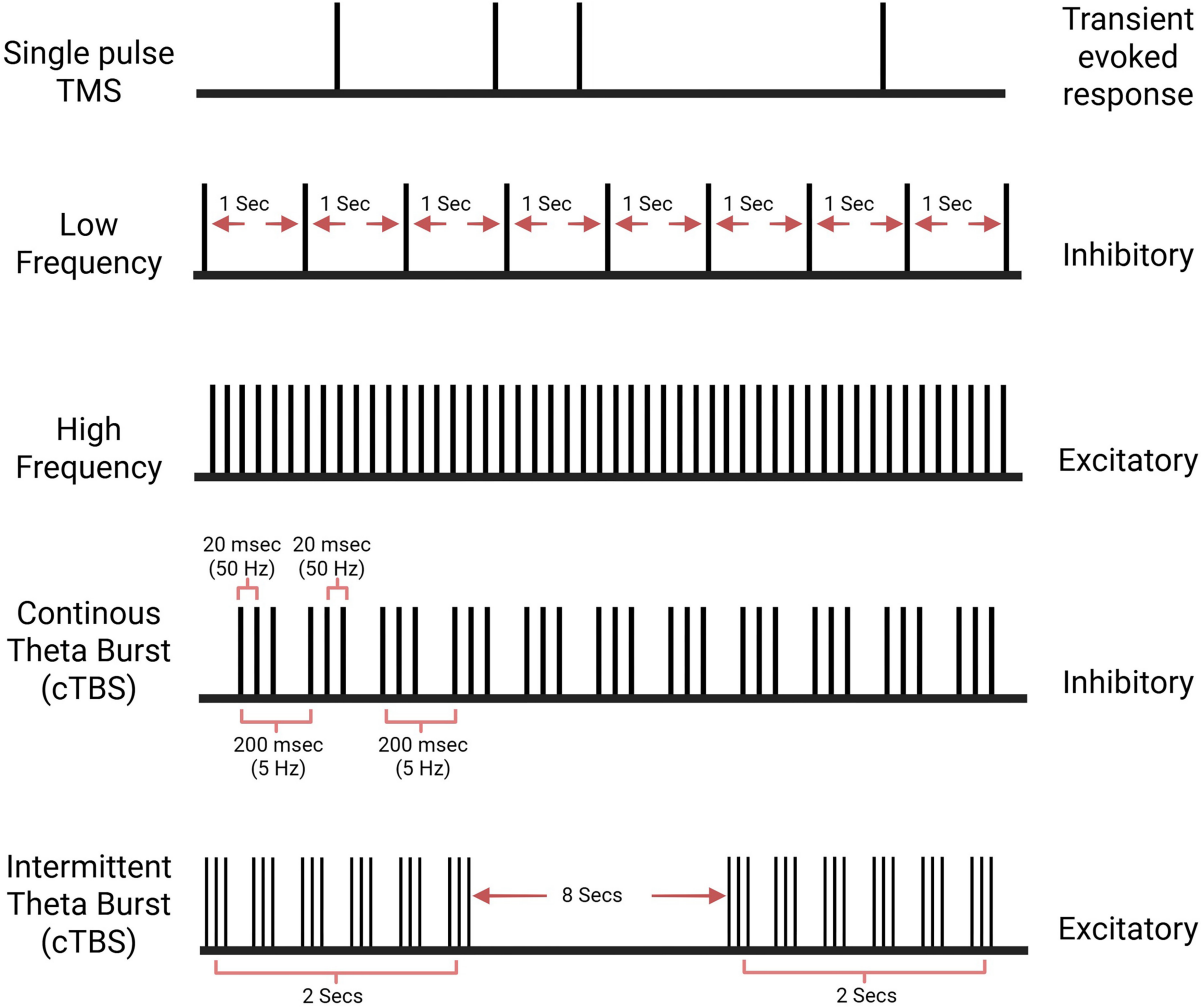
Pulse patterns of different repetitive transcranial magnetic stimulation (rTMS) protocols. Low-frequency rTMS is delivered at a rate of 1 Hz or lower and acts as inhibitory stimulation. High-frequency rTMS is delivered at a rate of 5 Hz or higher and acts as excitatory stimulation. Continuous theta-burst stimulation (cTBS) delivers a triplet burst of pulses continuously and acts as inhibitory stimulation. Intermittent theta-burst stimulation (iTBS) delivers a triplet burst of pulses intermittently, acting as excitatory stimulation. Created with BioRender.com.

TMS is a method to non-invasively modulate and measure cortical excitability that has been studied and applied since 1985 (Barker et al., 1985). In this section, we focus on the biological mechanisms underlying these effects, while clinical and cognitive outcomes are discussed separately. In general, single-pulse TMS, including paired-pulse variations, is used mainly to probe brain function, whereas rTMS is intended to induce longer-lasting changes in brain activity (Klomjai et al., 2015). These changes can be shaped by adjusting rTMS parameters to target specific neurophysiological outcomes. Key factors include pulse waveform, coil shape, stimulation intensity, target region, frequency, and pulse pattern (Caulfield and Brown, 2022; Figure 1).

### 3.1 Physiological basis of TMS effects on neuronal excitability

TMS exerts its physiological effects through interaction between the induced electric field and neuronal axons in the targeted cortex (Di Lazzaro and Rothwell, 2014; Li et al., 2017). This interaction initiates a complex response involving various types of neurons (Aberra et al., 2020). Research, including invasive rodent studies and human corticospinal tract measurements, shows that a single TMS pulse triggers a rapid sequence of synaptic activities in the motor cortex (Di Lazzaro and Rothwell, 2014; Li et al., 2017). Excitation of local neurons continues for milliseconds beyond the stimulus itself and is influenced by electric field direction (Di Lazzaro and Rothwell, 2014; Li et al., 2017). For rTMS, the physiological mechanisms underlying its after-effects are incompletely understood, but they are thought to parallel long-term potentiation (LTP) and long-term depression (LTD) processes described in animal models (Klomjai et al., 2015).

### 3.2 Understanding TMS mechanics using motor evoked potentials (MEP)

Since the introduction of TMS, Barker and his team initially applied it to the primary motor cortex region corresponding to the hand (Barker et al., 1985). Stimulating the M1-hand area and observing a corresponding motor response in the contralateral hand muscle provides compelling evidence of antegrade, trans-synaptic neural excitation along established neural pathways. This excitation not only impacts local cortical areas but also reaches interconnected cortical and subcortical regions, including motor neurons in the spinal cord. These motor responses can be easily detected through surface electrodes on the muscles of the contralateral hand (Groppa et al., 2012). Consequently, the motor cortex, particularly M1-hand, is often used as the primary cortical target in studies exploring TMS mechanisms (Rossini et al., 2015).

Locally, the motor threshold (MT) reflects reactivity of the motor pathway from cortex to peripheral muscle. More globally, it is also influenced by structural factors such as scalp-to-cortex distance, which affect the energy required to activate corticospinal neurons. This dual assessment provides insights into both local and global factors affecting motor cortex reactivity (Rossini et al., 2015). As an operational measure of cortical excitability, MT is defined as the lowest stimulus intensity that reliably evokes a motor evoked potential (MEP) of at least 50 μV peak-to-peak in at least 50% of trials (Rossini et al., 2015). This will determine the resting MT (rMT), typically assessed at rest; the active MT (aMT), assessed during slight tonic contraction, will be determined when the MEPs are greater than 100 μV (Rossini et al., 2015; Paulus et al., 2013). Current recommendations suggest calculating the MT based on 20 responses to enhance reliability (Goldsworthy et al., 2016). After determining the MT, most intervention protocols will use either subthreshold (< 100% of individual MT) or suprathreshold (> 100% of MT) stimulation intensity (Rossini et al., 2015).

A key neurophysiological feature of MEPs is the enhanced response observed with slight pre-activation of the target muscle (aMEP) compared with when the muscle is at rest (rMEP). This facilitation is attributable to changes at both spinal and cortical levels, that enhance transmission along the corticomotor pathways to the active muscle (Siebner et al., 2022). The MEP is recorded from the target muscle via surface electromyography (EMG) following stimulation of the M1-hand area. While recording it is straightforward, the neurophysiological processes underlying it are intricate. The MEP arises from the synchronized excitation of fast-conducting corticomotor neurons, which then transmit to the motor units in the target muscle (Siebner and Rothwell, 2003). However, this synchronization is incomplete, and differences across cortical, spinal, and muscular systems contribute to marked trial-to-trial variability of the MEP (Ziemann and Siebner, 2015). Additionally, phase cancelation can significantly reduce MEP amplitude, a phenomenon that can be largely mitigated using the triple stimulation technique (Magistris et al., 1999). Despite these complexities, MEP-based neurophysiological studies have been pivotal in deciphering the mechanisms by which TMS affects the M1 region.

Focusing on the M1 region, more complex TMS protocols, such as paired-pulse TMS (ppTMS), have been applied to investigate intracortical circuits relying on different neurotransmitter classes. In this protocol, two pulses are delivered within milliseconds of each other. The first, a conditioning pulse (CP; usually 80% of rMT), influences the response to the second, a suprathreshold test pulse (SP; generally at 120% of rMT). There are different ppTMS protocols, including short-interval intracortical inhibition (SICI), short-interval intracortical facilitation (SICF), and long-interval intracortical inhibition (LICI) (see Table 1). The protocols vary by the interstimulus interval; for example, if the interval between stimuli is around 2–3 ms, the CP should inhibit the response of the second one, and the amplitude of the MEP generated will be smaller than the one generated with the single-pulse TMS. This inhibitory response is observed even at stimulation intensities lower than those required to evoke a MEP in relaxed hand muscles (Ziemann et al., 1996). It is widely believed that the inhibitory effects are primarily mediated by GABAergic interneurons, which suppress excitatory TMS responses. Accordingly, the percentage reduction in MEP amplitude is often interpreted as an indirect measure of GABAergic activity. However, alternative mechanisms like “shunting inhibition,” attributed to activity-induced increases in transmembrane conductance, have also been proposed (Paulus and Rothwell, 2016).

**TABLE 1:** Summary of single- and paired-pulse transcranial magnetic stimulation (TMS) measures.

TMS variable	Definition	Plasticity changes	Aging	MCI	AD
Resting motor threshold (rMT)	Is the minimal intensity of stimulation necessary to elicit a MEP of 100 μV in the target muscles, in at least 50% of the attempts (Rossini et al., 2015)	Changes of rMT might represent an indirect measure of intrinsic plasticity in human motor cortex	rMT ⟷↑	rMT ↓	rMT ↓ aMT ↓
Motor evoked potential (MEP)	A MEP is the response recorded in the target muscle after a TMS stimulus (Rossini et al., 2015)	MEPs can be used to evaluate the integrity of cortico-spinal pathways; MEP amplitudes represent changes in the synaptic plasticity (LTP-like and LTD-like plasticity) and therefore changes of MEP amplitudes represent changes in the motor cortex plasticity			
Intracortical facilitation (ICF)	It can be elicited when a subthreshold (80% of rMT) cortical stimulation is followed by a suprathreshold (120% of rMT) stimulus at an intra-stimulus interval (ISI) of 6–30 ms, resulting in increased MEP amplitude (Rossini et al., 2015)	The physiological basis of ICF is still poorly understood. It is suggested that this form of facilitation involves glutamatergic circuits in M1. The modulation of intracortical excitability can induce plasticity; however, most of rTMS trials do not report significant changes in ICF	ICF ⟷	ICF ⟷	ICF ⟷↓
Intracortical inhibition (ICI/SICI)	It can be elicited when a subthreshold (80% of rMT) cortical stimulation is followed by a suprathreshold (120% of rMT) stimuli at an ISI of 1–6 ms, resulting in a decreased MEP amplitude (Rossini et al., 2015; Kujirai et al., 1993)	ICI can reflect the balance between inhibitory and excitatory networks in the motor cortex. It is believed to be related to neuronal refractoriness and post synaptic inhibition mediated by GABA receptors	SICI ⟷↓ LICI ⟷	SICI ⟷ LICI ⟷	SICI ⟷↓ LICI ⟷

When the interval between stimuli is around 10–12 ms, an MEP with a higher amplitude is expected. If the interval is approximately 6 ms, the MEP is expected to have the same amplitude as the one elicited by single-pulse TMS. In the intracortical facilitation (ICF) case, the percentage increase of MEP would be a measure of glutamatergic activity (Rossini et al., 2015). Thus, ICF is generally interpreted as reflecting the strength of excitatory intracortical synaptic interactions (Rossini et al., 2015; Ziemann et al., 2004)

### 3.3 TMS variations in aging, MCI, and AD

In aging individuals, TMS can be used to assess changes in various neurophysiological parameters related to the motor cortex function. Studies have reported an increase in rMT in older adults, suggesting changes in cortical atrophy and structural alterations with age (Bhandari et al., 2016; Levin et al., 2011; Peinemann et al., 2001; Rossini et al., 1992). However, some studies have found no change in rMT in older adults when compared with younger individuals (Oberman and Benussi, 2023). This discrepancy may be attributed to individual patterns of cortical atrophy, more common in older adults, which can increase coil-to-cortex distance, and these measures are usually not assessed in these cross-sectional trials (Julkunen et al., 2012; McConnell et al., 2001). Another interpretation of the inconsistent age-related differences in rMT suggests that the MT undergoes different stages during the transition from young adulthood to aging (Gomes-Osman et al., 2018). Shibuya et al. (2016) found that age-related changes in rMT follow a quadratic curve, increasing until age 50 and decreasing thereafter; longitudinal studies are required to confirm this hypothesis (Shibuya et al., 2016).

As the brain ages, it undergoes multiple structural and vascular changes. These include cerebral atrophy, gray and white matter alterations, ventricular enlargement, sulcal widening, reduced vessel elasticity, increased arterial stiffness, and diminished cerebrovascular reactivity (Peters, 2006; Burke and Barnes, 2006; Zimmerman et al., 2021). This vascular aging is associated with increased cerebral blood flow pulsatility, impaired autoregulation, white matter hyperintensities, gray matter volume loss, and cognitive decline (Tarumi and Zhang, 2018; Zimmerman et al., 2021). Beyond rMT, the stimulus-response relationship may also change with age. Older adults may require higher stimulation intensities to achieve maximal MEPs (Pitcher et al., 2003), possibly because fewer motor neurons are recruited or because their firing is less synchronized. In contrast, findings from ppTMS in aging remain inconsistent, making it challenging to determine the status of GABAergic neurotransmission. Among intracortical measures, short-afferent inhibition (SAI) shows the most consistent age-related alterations, with studies reporting decreased SAI in older adults, indicating a progressive cholinergic activity dysfunction (Dumas and Newhouse, 2011). For more details, we direct readers to a recent review by Di Lazzaro and colleagues (Di Lazzaro et al., 2021).

In AD, increased cortical excitability is observed by decreased MT, especially in the early stages (Hoeppner et al., 2012; Liepert et al., 2001; Di Lazzaro et al., 2002) of the disease, and when compared with older adults without cognitive impairment (Di Lazzaro et al., 2021). As the disease progresses, this hyperexcitability changes in advanced stages, likely due to brain atrophy and reduced neural fiber density. Findings are not entirely consistent, as some researchers report there is no difference in MT between healthy older adults, individuals with early-onset dementia, and those with frontotemporal dementia (Pierantozzi et al., 2004).

In terms of cortical plasticity, patients with AD show a reduced response to stimulation compared with healthy individuals. Certain forms of plasticity, like long-term depression induced by low-frequency rTMS, appear more resilient in AD. Cortical inhibition is also impaired, as shown by reduced including short- and long-interval intracortical inhibition (Hoeppner et al., 2012; Liepert et al., 2001; Di Lazzaro et al., 2002; Antczak et al., 2021). SAI, performed to indirectly assess central cholinergic activity, is decreased in AD (Di Lazzaro et al., 2002), and can be transiently restored by the administration of levodopa (Martorana et al., 2008). Interestingly, acetylcholinesterase inhibitors used for AD treatment enhance cortical inhibition without affecting MT (Di Lazzaro et al., 2005a,^2006^).

Like AD patients, those with vascular dementia (VaD) also exhibit reduced rMT (Di Lazzaro et al., 2006; Khedr et al., 2020). However, findings on SAI are less consistent: some show impairment (Nardone et al., 2014), and others suggest it remains unchanged, likely reflecting the overlap between AD and VaD (Pennisi et al., 2011). SAI impairment is more prominent in cerebral autosomal-dominant arteriopathy with subcortical infarcts (CADASIL), a genetic form of VaD that is often accompanied by decreased ICF (Mizuno et al., 2020; Nardone et al., 2014). Of note, SAI impairment in CADASIL is not reversed by dopamine, unlike in AD (Nardone et al., 2014), indicating distinct mechanisms underlying cholinergic transmission impairment. In CADASIL, this may reflect infarcts affecting cholinergic pathways rather than a primary neurodegenerative process (Nardone et al., 2014). In most VaD studies, other TMS parameters appear unchanged (Mizuno et al., 2020; Nardone et al., 2014).

Most of the current literature is focused on the disruption of neurotransmitter systems that are associated with cognitive impairment, such as GABA, glutamate, and acetylcholine (Xu et al., 2020; Nava-Mesa et al., 2014). Recent studies demonstrate that cerebrovascular dysfunction plays a pivotal role in neurodegenerative disease and could precede alterations seen in neurons (Zhu et al., 2022; Ungvari et al., 2018). In this context, growing evidence shows further decreases in neurovascular coupling (NVC) responses in older adults with cognitive impairment compared with age-matched controls (Mukli et al., 2023; Owens et al., 2024; Ruan et al., 2023). These alterations should be considered when designing TMS protocols aimed at enhancing cognitive performance in healthy older adults versus those with cognitive impairment. The apparent paradox that cognitive impairment may correlate with reduced rMT, despite brain atrophy and increased cortex-to-coil distance that would typically elevate rMT (De Carvalho et al., 1997; Di Lazzaro et al., 2004, 2002), may reflect metabolic and functional connectivity changes. This remains a hypothesis for future study.

### 3.4 Modulation of neuroplasticity through TMS

**FIGURE 2:**
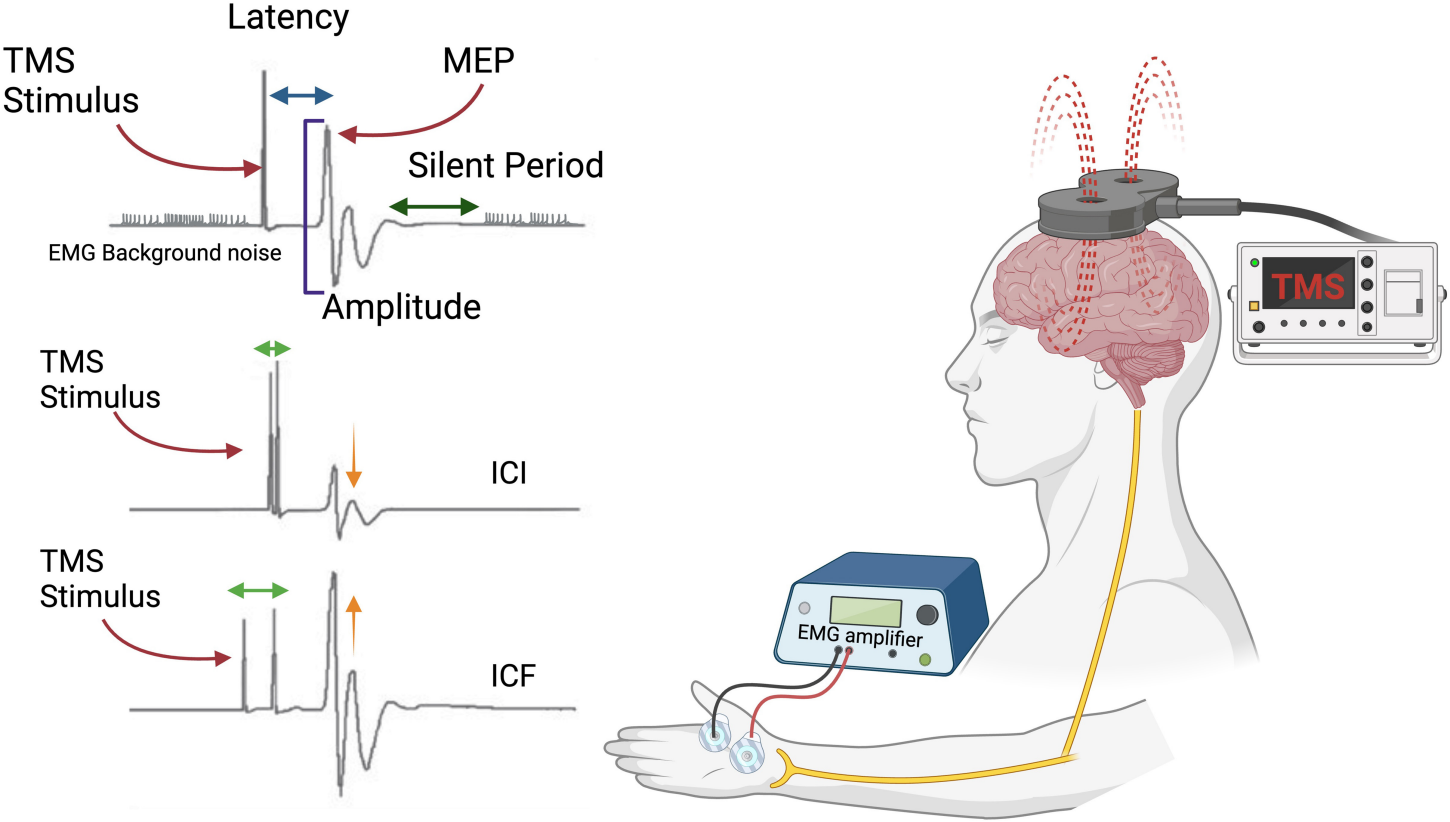
Schematic representation of transcranial magnetic stimulation (TMS) over the motor cortex. TMS produces a rapidly changing magnetic field that permeates the skull, generates an electric field in the underlying tissue, and, ultimately, leads to the depolarization of cortical neurons. When applied at sufficiently high intensities, this stimulation of motor cortical regions elicits motor-evoked potentials (MEPs) in peripheral muscles associated with these regions. These MEPs can be quantified through electromyography. EMG, electromyography; ICF, intracortical facilitation; ICI, intracortical inhibition. Created with BioRender.com.

rTMS delivers repeated pulse trains of magnetic fields at consistent intensity and defined frequency, modulating cortical excitability to promote either inhibition or facilitation. Conventionally, low-frequency rTMS (LF < 5 Hz) is considered inhibitory, whereas high-frequency rTMS (HF > 5 Hz) is considered facilitatory (Pell et al., 2011; Fitzgerald et al., 2006). However, this binary classification is likely oversimplified, particularly in neurodegenerative disease (Hussain and Freedberg, 2025). In AD, cortical disinhibition related to GABAergic impairment may cause nominally “inhibitory” protocols to paradoxically act as restorative interventions. Rather than simply suppressing neuronal activity, these protocols may help re-establish excitation-inhibition balance and stabilize network dynamics (Bashir et al., 2022). In addition to these conventional protocols, theta-burst stimulation (TBS) can be applied, with its effect on excitability determined by the interstimulus intervals of the bursts. TBS consists of three very short high-frequency bursts, which can decrease cortical excitability when delivered continuously (cTBS) and increase it when delivered intermittently (iTBS) (Di Lazzaro et al., 2005b; Huang et al., 2005; Figure 2). The final effects of rTMS depend on the stimulation frequency, baseline brain activity, and regulatory mechanisms that maintain brain excitability. One such mechanism is homeostatic plasticity, which helps neural networks stabilize activity levels in response to prior changes in neuronal activity and may contribute to interindividual variability in stimulation response. rTMS effects can also be altered by stimulus intensity, commonly defined as a percentage of the individual’s rMT (Di Lazzaro et al., 2005b; Huang et al., 2005).

The basic principle of rTMS is that magnetic stimulation generates transient fields that depolarize neurons, ultimately leading to neurotransmitter release at the post-synaptic cleft and excitation of circuits involved in synaptic plasticity. HF-rTMS stimulation has been shown to increase local blood flow (Hernandez-Martin et al., 2019), consistent with the relationship between neuronal activity and perfusion (Ward, 2016). The long-lasting therapeutic effects of rTMS are thought to involve LTP-like and LTD-like plasticity (Hoogendam et al., 2010). HF-rTMS and TBS can elicit LTP-like effects (Duffau, 2006), particularly when presynaptic activity is followed within tens of milliseconds by postsynaptic activation. Conversely, LF-rTMS can induce LTD, where the postsynaptic activation precedes presynaptic stimulation within a similar interval (Dan and Poo, 2006). The time-dependent effect changes in neuronal response are not observed when the interval exceeds 100 ms (Bi and Poo, 1998; Awiszus et al., 1999).

In humans, 1 Hz magnetic stimulation led to a decrease in induced muscle responses, reflecting LTD-like effects (Wassermann, 1998; Chen et al., 1997a; Touge et al., 2001; Maeda et al., 2002; Muellbacher et al., 2002). A 15-min LF-TMS session at 0.9 Hz (800 pulses) with a stimulation intensity of 115% of the rMT resulted in a 20% decrease in the induced muscle response that lasted 15 min beyond the end of the stimulation (Chen et al., 1997a). Further, an increase in cortical excitability is observed after HF stimulation of the M1. In one of the first rTMS studies, Pascual-Leone and collaborators showed a 50% increase in the induced muscle response after 20 HF-rTMS pulses at an intensity of 150% of the rMT (Pascual-Leone et al., 1994). Although these studies support the widely accepted view that HF-rTMS induces LTP-like effects and LF-rTMS induces LTD-like effects, this framework has important limitations (Chervyakov et al., 2015). Its application in practice is more complex, as the specific outcomes of brain stimulation are inherently influenced by a multitude of factors (Ziemann and Siebner, 2015).

The same TMS protocol can produce contrasting physiological responses when applied to different cortical regions (Ganzer et al., 2013), or to different individuals (Ziemann and Siebner, 2015; Goldsworthy et al., 2021). Even within the same brain region, the effects can vary based on the ongoing neural activity at the time of the stimulation (Jannati et al., 2023). These observations of variability and state-dependent responses highlight the intricate nature of TMS action and suggest that its effects may depend on baseline physiological and network characteristics, with implications for how stimulation is applied across individuals and disease stages (Ganzer et al., 2013; Goldsworthy et al., 2021; Jannati et al., 2023; Hoogendam et al., 2010; Caulfield and Brown, 2022).

rTMS may also exert additional mechanistic effects by altering cell processes, including the expression of receptors and neuromodulators (Hausmann et al., 2000; Chervyakov et al., 2015). Animal studies suggest that rTMS can decrease β-adrenoreceptors in frontal and cingulate cortices, increase NMDA receptors in the ventromedial thalamus, amygdala, and parietal cortex (Chervyakov et al., 2015; Lisanby and Belmaker, 2000), and raise nitric oxide (NO) and cyclic guanosine monophosphate (cGMP) levels in the cortex and hippocampus (Jannati et al., 2023).

Translating these cellular and receptor-level effects into consistent human outcomes depends on precise stimulation parameters, including intensity. One often overlooked issue is the common practice of setting stimulation intensity according to the individual MT. The M1 has unique structural and functional characteristics, including fast-conducting pyramidal neurons that generate a measurable MEP (Geyer et al., 2000; Spampinato et al., 2023). Activation triggers descending volleys along the corticospinal tract, producing a measurable MEP. In contrast, cognitively relevant but “silent” cortical areas, such as the left dorsolateral prefrontal cortex (DLPFC), do not produce these direct muscle outputs. Consequently, the MT determined over M1 serves as a pragmatic proxy for global cortical excitability rather than a precise measure for non-motor targets. Empirical studies comparing rMTs or aMTs with phosphene thresholds generally find no correlation (Boroojerdi et al., 2002; Antal et al., 2003), with some exceptions (Deblieck et al., 2008). While imperfect, using the MT accounts for global anatomical variables, such as skull thickness and scalp-to-cortex distance, which affect the electric field reaching the brain regardless of the target region. To overcome the limitations of this motor-based surrogate, future protocols may increasingly rely on TMS combined with electroencephalography (TMS-EEG). This technique allows direct assessment of cortical reactivity in non-motor areas through TMS-Evoked Potentials (TEPs), offering a more physiological way to dose stimulation in cognitive networks.

### 3.5 Potential mechanisms for TMS effects in AD

TMS may modulate the activity of neural circuits disrupted by AD pathology, thereby improving cognitive function. These mechanisms highlight the potential of TMS to address underlying neurobiological changes in AD, offering hope for this challenging condition.

#### 3.5.1 Neurogenic and neuroprotective effects of rTMS in AD

In AD patients, dysregulation and altered expression of neurotrophic factors (NTFs) such as nerve growth factor (NGF), brain-derived neurotrophic factor (BDNF), glial cell line-derived neurotrophic factor, and ciliary neurotrophic factor have been observed in different brain regions (Gezen-Ak et al., 2013; Lee et al., 2009). These changes are thought to contribute to AD neurodegeneration and are linked to cognitive decline (Budni et al., 2015; Gao et al., 2022; Laske et al., 2011). rTMS may have the potential to regulate NTF expression in the AD brain, thereby promoting neuronal survival and differentiation (Bashir et al., 2022).

Studies have shown an upregulation of BDNF, neuronal nuclear protein, and neuroepithelial stem cell protein in the hippocampus and cerebral cortex following HF-rTMS (Velioglu et al., 2021; Bashir et al., 2022). BDNF plays a crucial role in memory formation and synaptic plasticity via the BDNF-tropomyosin receptor kinase B (TrkB) signaling pathway, and its deficits are associated with AD (Bashir et al., 2022). Interestingly, studies report the influence of the BDNF Val66Met gene polymorphism on individual response to stimulation (Abellaneda-Perez et al., 2022). The Val66Met polymorphism has been associated with various neurophysiological and neuropsychiatric outcomes. For instance, individuals carrying the methylated allele often show differences in brain structure, including hippocampal volume and function (Pezawas et al., 2004; Egan et al., 2003). Abellaneda-Perez and colleagues used a rTMS-induced memory impairment protocol and showed that rTMS led to reduced memory performance only in the Val/Val allele carriers (Abellaneda-Perez et al., 2022). This group also exhibited increased functional magnetic resonance imaging (fMRI) brain activity during memory recognition, particularly in frontal regions, with this activity positively correlating with cognitive performance. These findings suggest that the BDNF Val66Met gene polymorphism, known for its significant effect on neuroplasticity, modulates the impact of rTMS at both cognitive and brain network levels.

The effects of rTMS on NGF, which is essential for the growth and survival of neuronal populations, have also been documented. Studies have demonstrated that LF-rTMS can upregulate NGF content in AD models (Choung et al., 2021). Chen and colleagues observed that both high and low frequencies of rTMS regulated brain levels of NTFs, including BDNF and NGF, with increased levels correlating with increased frequency (Chen et al., 2019). Interestingly, rTMS applications tend to decrease BDNF levels in healthy volunteers, contrasting with its effects in AD (Gaede et al., 2014; Schaller et al., 2014).

Besides the decrease in BDNF and other neurogenic factors, there’s a noted downregulation of apoptosis-inhibiting factors like Bcl-2, alongside an upregulation of pro-apoptotic elements such as Bax and cleaved caspase-3 (Paradis et al., 1996), which can lead to excessive neuronal loss in AD (Chi et al., 2018). rTMS has been shown to inhibit neuronal cell death by regulating various cell signaling pathways associated with enhanced apoptosis. For example, in AD mouse models, both 1 Hz and 10 Hz rTMS treatments resulted in increased Bcl-2 expression and reduced levels of Bax and cleaved caspase-3, indicating a suppression of apoptosis (Chen et al., 2019). Similarly, 1 Hz rTMS in a rat model of VaD increased Bcl-2 expression and suppressed Bax expression (Yang et al., 2015). In an animal study involving middle cerebral artery occlusion, 10 Hz rTMS treatment significantly upregulated Bcl-2 and decreased Bax and TUNEL-positive cells in the ischemic hippocampus (Guo et al., 2017). By modulating Bcl-2 and Bax, rTMS may suppress apoptotic signaling and thereby support functional recovery and protective mechanisms in AD (Guo et al., 2017). While further research is needed to fully understand TMS’s impact on apoptosis in AD pathology, these findings position TMS as a promising approach for clinical AD treatment.

#### 3.5.2 Oxidative stress and rTMS in AD

Oxidative stress is a key factor and major contributor to the progression of AD (Ionescu-Tucker and Cotman, 2021). It is characterized by an imbalance in cellular redox status, increased production of reactive oxygen species (ROS), and impaired antioxidant defense, leading to cellular dysfunction and damage, particularly in neurons (Butterfield and Halliwell, 2019). Oxidative stress is associated with AD’s histopathological hallmarks, such as amyloid plaques and neurofibrillary tangles (Butterfield and Halliwell, 2019; Ionescu-Tucker and Cotman, 2021).

rTMS has shown potential in modulating and balancing not only BDNF levels but also oxidative stress, suggesting its beneficial antioxidant effects in AD. A study by Velioglu and colleagues reported that 20 Hz rTMS applied to the lateral parietal cortex in AD patients increased BDNF and total antioxidant status while reducing total oxidant status and oxidative stress index (Velioglu et al., 2021). In addition, oxidative stress is intricately linked with BDNF, and oxidative stress markers could potentially serve as biomarkers for AD prognosis (Durmaz et al., 2018). Using a model of oxidative damage in the brains of Wistar rats, TMS was found to partially prevent or reverse these oxidative changes. The reduction in oxidative stress markers and the preservation of enzymatic activities implicated in oxidative defense suggest that TMS can potentially protect neuronal integrity and maintain synaptic health (Tunez et al., 2006).

Despite these promising findings, there is a significant gap in research regarding TMS’s impact on oxidative stress, antioxidant defense systems, and total oxidant/antioxidant status in AD. This gap highlights the need for further studies on how TMS affects oxidative stress and whether this contributes to its therapeutic potential in neurodegenerative disorders (Wu et al., 2022).

#### 3.5.3 Neurovascular coupling and hemodynamic regulation

While traditionally viewed through the lens of synaptic plasticity, the therapeutic scope of rTMS extends to the neurovascular unit. Cognitive function relies on the precise temporal coordination between neuronal activity and cerebral blood flow via NVC (van Dinther et al., 2024). This coupling is frequently impaired in aging and AD, creating a metabolic mismatch that accelerates neurodegeneration (Tarantini et al., 2017; Attwell et al., 2010; Toth et al., 2017). rTMS has been shown to induce hemodynamic changes that parallel neuronal activation, modulating local cerebral blood flow and potentially supporting blood-brain barrier integrity (Näsi et al., 2011; Paus et al., 1998; Zhang et al., 2022; Zong et al., 2020). By engaging the neurovascular unit, rTMS may help restore metabolic support to hypoperfused neural networks. This may counter the vascular dysregulation that often precedes overt cognitive decline. Accordingly, the efficacy of TMS in AD may depend not only on synaptic reorganization but also on restoration of healthy neurovascular responses.

#### 3.5.4 Neurotransmitters in AD and rTMS

AD’s onset adversely affects the metabolism and levels of various neurotransmitters crucial for cognitive control, learning, and memory development, such as dopamine, glutamate, aspartate, and GABA. Neurotransmitter alterations lead to synaptic dysfunction, cognitive impairment, and memory deficits, with a marked reduction in neurotransmitters and receptors in patients with AD (Yang et al., 2023). HF (20 Hz) and LF (1 Hz) rTMS increased dopamine levels in the hippocampus, with the expression of dopamine receptor 4 also elevated following 1 Hz rTMS in the hippocampus and cerebral cortex of the AD brain (Choung et al., 2021). Similar increases in dopamine levels have been observed in healthy individuals following deep TMS therapy (Malik et al., 2018). The loss of dopamine and its receptors is frequently reported in AD, contributing to motor and cognitive decline (Pan et al., 2019; D’Amelio et al., 2018).

Another critical molecule in synaptic transmission is the N-methyl-D-aspartate receptor (NMDAR) (Zhang Y. et al., 2016). In AD, Aβ plaques induce excessive calcium influx through NMDARs, leading to synaptic dysfunction and neuron death (Kodis et al., 2018). TMS has been shown to regulate NMDAR expression, which is downregulated in AD. LF-rTMS can increase the expression of NMDAR and its subunits (NR1, NR2A, NR2B) in the hippocampus, facilitating LTP and memory formation (Tan et al., 2013). An increase in NMDAR and vascular endothelial growth factor expression was also observed in a rat model of VaD following rTMS treatment. On the other hand, NMDA receptor modulators like memantine and d-cycloserine can influence/enhance the effects of rTMS due to their role in facilitating LTP (Huang et al., 2007; Teo et al., 2007). This interaction highlights the complexity of rTMS effects and the need to consider drug interactions when planning. *In vitro* experiments also showed the potential of TMS to enhance plasticity. TBS of SH-SY5Y *human* neurons cells significantly enhanced expression of plasticity genes *NTRK2* and *MAPK9* 24 h after iTBS compared with sham TBS (Thomson et al., 2020).

#### 3.5.5 Integrative mechanistic framework of TMS effects

**FIGURE 3:**
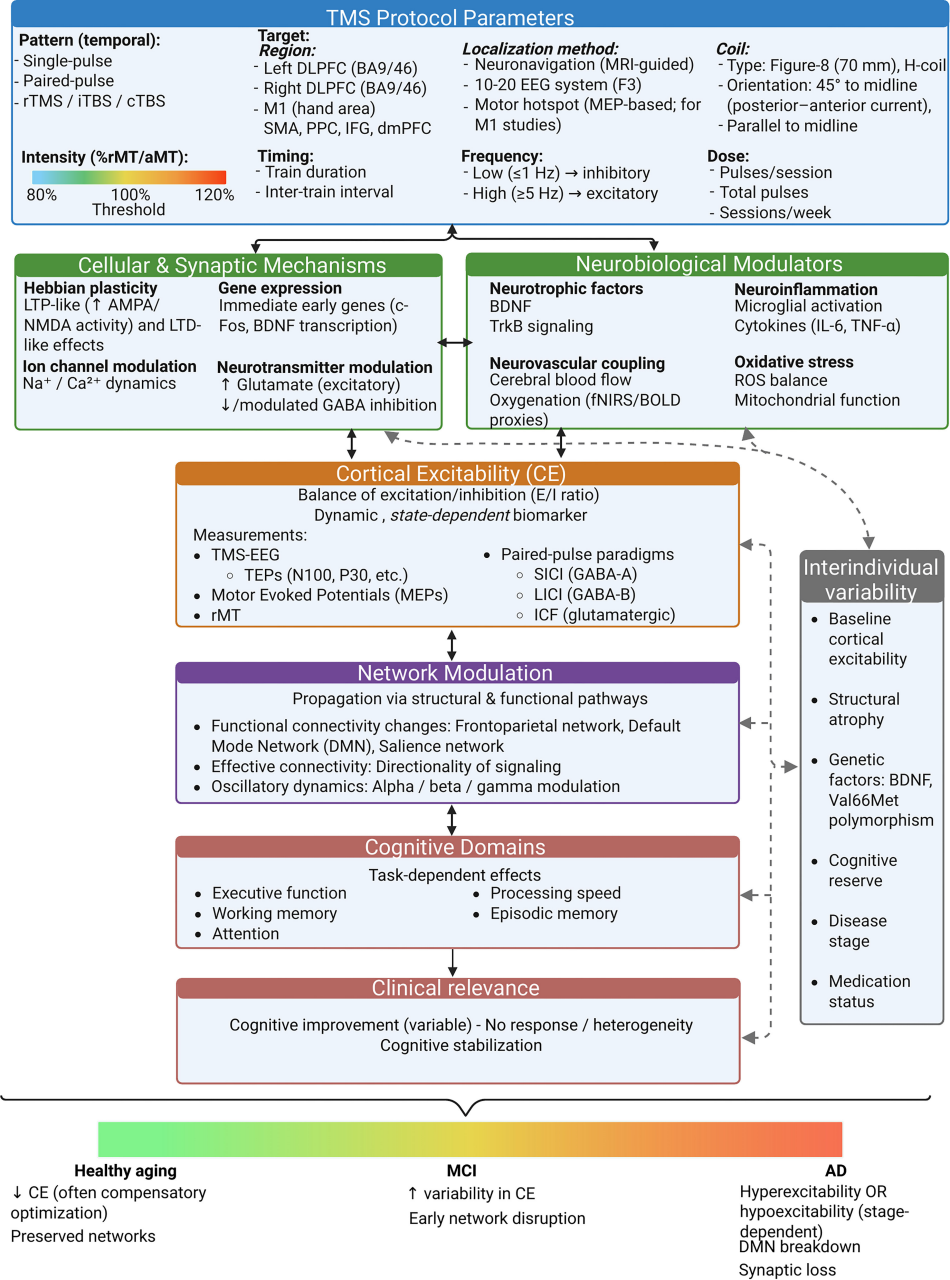
Integrative framework of transcranial magnetic stimulation (TMS) effects across aging and cognitive decline. TMS modulates cortical excitability through stimulation parameters such as frequency, intensity, and cortical target. These effects engage in synaptic plasticity, neurotransmitter systems, neurotrophic signaling, and neurovascular dynamics, and converge at the level of large-scale brain networks. Resulting changes in functional connectivity influence cognitive domains across healthy aging, MCI, and AD, with effects shaped by individual brain state and disease stage. TMS, transcranial magnetic stimulation; rTMS, repetitive transcranial magnetic stimulation; MCI, mild cognitive impairment; AD, Alzheimer’s disease; DLPFC, dorsolateral prefrontal cortex; DMN, default mode network; NVC, neurovascular coupling; LPC, lateral parietal cortex; NMDA, N-methyl-D-aspartate; BDNF, brain-derived neurotrophic factor; fNIRS, functional near-infrared spectroscopy; EEG, electroencephalography. Created with BioRender.com.

Although the above mechanisms are often discussed separately, the biological effects of TMS likely emerge from their interaction across multiple levels of neural organization. Modulation of synaptic plasticity, neurotransmitter systems, neurotrophic signaling, and neurovascular dynamics can collectively influence cortical excitability and the functional connectivity of distributed brain networks (Abellaneda-Perez et al., 2022; Barker et al., 1985; Bashir et al., 2022; Chervyakov et al., 2015; Cirillo et al., 2017; Duffau, 2006; Ilmoniemi et al., 1997; Julkunen et al., 2012; López-Alonso et al., 2014; Miniussi et al., 2010; Nord, 2021; Owens et al., 2022; Siebner et al., 2022; Ziemann et al., 2015). Together, these changes may reshape communication between regions that support cognitive outcomes. Consequently, cognitive outcomes after stimulation are better understood as network-level effects shaped by coordinated changes in cortical excitability and inter-regional communication, rather than by isolated local modulation (Di Fazio et al., 2026; Palermo et al., 2025; Zadey et al., 2021; Figure 3).

## 4 Effects of rTMS on cognitive outcomes

Having outlined the underlying biological mechanisms, the following sections reviews the evidence on cognitive and clinical outcomes across healthy aging, MCI, and Alzheimer’s Disease (Table 2).

**TABLE 2:** Conceptual synthesis of stimulation strategies and cognitive targets across the healthy aging and cognitive decline continuum.

Category	Healthy aging	MCI	Alzheimer’s disease
Network target	Left and right DLPFC	Left DLPFC (primary); emerging targets include lateral parietal cortex (LPC)	Left DLPFC (most common); multisite cortical targets (e.g., temporal, parietal regions)
Typical stimulation strategies	High-frequency rTMS (left DLPFC); low-frequency rTMS (right DLPFC); task-based stimulation paradigms (Barr et al., 2009; Gaudeau-Bosma et al., 2013; Kim et al., 2012; Patel et al., 2020; Pearce et al., 2014)	High-frequency rTMS over left DLPFC; multisite stimulation approaches; combined protocols with cognitive tasks (Chu et al., 2021; Šimko et al., 2022; Wang et al., 2020)	High-frequency rTMS over left DLPFC; multisite protocols (e.g., Neuro-AD); sequential stimulation paradigms (Ahmed et al., 2012; Chu et al., 2021; Dong et al., 2018; Rutherford et al., 2015; Li et al., 2023; Turriziani et al., 2019)
Cognitive domains addressed	Memory, attention, executive function, processing speed	Global cognition, memory (most consistent), domain-specific effects	Memory, language, global cognition
Key mechanistic rationale	Modulation of cortical excitability and functional connectivity; support of compensatory recruitment (e.g., bilateral PFC engagement, HAROLD model) (Cabeza, 2002; Manenti et al., 2011; Meinzer et al., 2013; Rossi et al., 2004)	Modulation of frontoparietal and default mode network interactions; partial preservation of network integrity allows plasticity induction; distributed network engagement may enhance effects (Liston et al., 2014; Meinzer et al., 2013; Wang et al., 2014)	Modulation of disrupted large-scale networks; effects on neurotransmitter systems (dopaminergic, NMDA-related pathways); attempts to engage distributed network dysfunction (Choung et al., 2021; D’Amelio et al., 2018; Zhang L. et al., 2016)
Translational considerations	Effects are often modest and inconsistent; baseline cognitive reserve and preserved function may limit observable benefit; protocol effects may depend on task engagement and baseline performance (Cabeza et al., 2002; Patel et al., 2020)	Heterogeneous outcomes across studies; differences in cognitive outcome definitions and stimulation parameters limit comparability; small sample sizes and short follow-up are common limitations (Chu et al., 2021; Šimko et al., 2022; Wang et al., 2021). Baseline increased cortical excitability and reduced LTP-like plasticity may influence response to stimulation (Buss et al., 2020; Chou et al., 2022)	Greater network disruption and heterogeneity in response; variability in disease stage, protocol design, and outcome measures; limited evidence of sustained clinical benefit; small cohorts and short follow-up limit interpretation (Ahmed et al., 2011; Alcalá-Lozano et al., 2018; Dengler et al., 2024; Fazio et al., 2025; Hu et al., 2022; Lv et al., 2023; Yao et al., 2022). Increased cortical excitability, reduced inhibition and impaired LTP-like plasticity may shape response to stimulation (Chou et al., 2022; Di Lorenzo et al., 2019).

Over the past decade, rTMS has gained substantial recognition as a promising neuromodulatory technique for enhancing cognitive outcomes across aging and clinical populations (Wang et al., 2021; Zhang et al., 2021; Bashir et al., 2022; Menardi et al., 2022; Šimko et al., 2022; Wu et al., 2022). Evidence from clinical trials suggests that rTMS can improve multiple cognitive domains in healthy older adults as well as in individuals with MCI, and dementia, including vascular dementia (VaD) and Alzheimer’s disease (AD) (Kim et al., 2019). Recent studies report positive effects on both general cognition and domain-specific outcomes such as memory, attention, executive function, and language (Teselink et al., 2021; Chu et al., 2021; Wang et al., 2021; Patel et al., 2020; Dong et al., 2018; Wang et al., 2020; Lin et al., 2019; Zhang et al., 2021; Cheng et al., 2018; Chou et al., 2020; Šimko et al., 2022). Effect sizes range from moderate to large, including notable benefits in AD populations (Wang et al., 2020) and sustained cognitive gains following rTMS in individuals with MCI and AD (Chu et al., 2021). Collectively, this literature highlights rTMS as a clinically relevant tool for modulation of cognitive outcomes, though its effects vary according to population and disease severity.

### 4.1 Impact of TMS on cognition in healthy older adults

Research in healthy aging populations has increasingly focused on TMS as a tool to enhance cognitive outcomes. Targeted stimulation protocols have been associated with improvements in memory, attention, executive functioning, and processing speed, likely through modulation of cortical excitability and plasticity-related mechanisms (Barr et al., 2009; Gaudeau-Bosma et al., 2013; Kim et al., 2012; Pearce et al., 2014). However, the effectiveness of these interventions depends on baseline age-related differences in cortical excitability and network recruitment, which themselves translate into declines in memory, executive function, and processing speed, with some changes potentially compensatory (Jannati et al., 2023; Manenti et al., 2011; Rossi et al., 2004; Ferreri et al., 2017).

Despite the promising reports, the meta-analytic literature on this topic remains limited. Only one meta-analysis has evaluated offline rTMS in healthy older adults (Patel et al., 2020). It found generally small or negligible effects across cognitive domains, with modest benefits in executive function following excitatory stimulation of the DLPFC (mostly left) and slight enhancements in episodic memory and visual perception after inhibitory stimulation; no reliable changes were observed in working memory performance (Patel et al., 2020). Although these results suggest limited effects of rTMS in the healthy aging brain, interpreting them requires consideration of the substantial heterogeneity in both cognitive aging and baseline physiology. Some older adults perform comparably to younger adults on memory tasks, suggesting a degree of resilience or compensatory capacity that may reduce the observable impact of neuromodulation (Cabeza et al., 2002).

rTMS applied to the left DLPFC may enhance memory recall and attentional performance, suggesting that targeted stimulation selectively improves specific cognitive systems (Hauer et al., 2019). Older adults may experience gains comparable to those reported in younger sample individuals, although the neurocognitive mechanisms underlying these improvements may differ with age (Rossi et al., 2004; Manenti et al., 2011). Evidence suggests that TMS may help restore or stabilize more “youth-like” neural activation patterns through functional connectivity modulation (Meinzer et al., 2013). Episodic memory provides an illustrative example, as neuroimaging studies have documented age-related changes in prefrontal cortex (PFC) activation during memory encoding and information retrieval (Cabeza et al., 1997; Grady et al., 1998; Manenti et al., 2012). Earlier work in younger adults identified a hemispheric specialization within the DLPFC, with the left hemisphere supporting encoding and the right supporting retrieval (Rossi et al., 2001). The predominance of the left DLPFC’s effect during encoding was not diminished in older individuals, implying its enduring importance for encoding across the lifespan. These observations suggest that the neural correlates of episodic memory retrieval evolve with aging, and engagement of both DLPFC regions in older adults may serve a compensatory function to support episodic memory performance (Rossi et al., 2004). The findings have led to the HAROLD (Hemispheric Asymmetry Reduction in Older adults) model, which postulates bilateral PFC involvement in both the encoding and retrieval phases in aging individuals (Cabeza et al., 2002; Cabeza, 2002).

An influential application of this model was demonstrated in a study by Rossi et al. (2004), who applied rTMS over the left or right DLPFC during encoding and retrieval of visual stimuli in healthy older adults (Rossi et al., 2004). In younger adults, right-hemisphere stimulation disrupted retrieval more strongly than left-hemisphere stimulation, consistent with expected lateralization (Rossi et al., 2004). In older adults, by contrast, stimulation of either hemisphere disrupted retrieval, supporting the idea of reduced hemispheric specialization with age (Rossi et al., 2004). Importantly, left-hemisphere involvement in encoding remained dominant across age groups, highlighting its enduring importance for encoding processes throughout the lifespan (Rossi et al., 2004).

Compensatory mechanisms may also underlie the beneficial effects of rTMS, where it significantly improved associative memory in older adults, particularly those with subjective memory complaints, lower-range memory performance and impaired recollection-based retrieval (Solé-Padullés et al., 2006; Cui et al., 2020). These cognitive gains have been associated with increased activation in the right prefrontal cortex during post-treatment fMRI sessions, relative to baseline (Solé-Padullés et al., 2006). Such findings reinforce the notion that neural compensation, reflected in expanded or reorganized activation patterns, may play a critical role in preserving cognitive function with advancing age and may be strengthened by targeted neuromodulation. While these findings provide insight into physiological aging, the application of TMS in pathological cognitive decline introduces additional complexity.

### 4.2 Impact of TMS on cognition in individuals with cognitive decline and MCI

Growing evidence suggests that TMS may offer therapeutic benefits for individuals with cognitive decline or MCI. Some in MCI populations report that rTMS produces measurable improvements in both general cognition and memory relative to sham stimulation. Other studies describe more domain-specific effects, with improvement limited primarily to memory rather than broader cognition (Šimko et al., 2022; Teselink et al., 2021; Chu et al., 2021; Wang et al., 2021; Dong et al., 2018; Wang et al., 2020; Lin et al., 2019; Zhang et al., 2021; Cheng et al., 2018; Chou et al., 2020). Evidence further suggests that individuals with MCI exhibit increased cortical excitability and reduced LTP-like plasticity, and these changes may be particularly relevant to memory-related outcomes (Buss et al., 2020; Chou et al., 2022). However, not all findings point in a positive direction, and some report no significant benefits for individuals with MCI (Chu et al., 2021). This variability may reflect both study-level differences and physiological heterogeneity in MCI.

A central challenge in this field is the selection of optimal stimulation targets. Most rTMS studies in MCI focus on the left DLPFC, based largely on work in major depressive disorder (Liston et al., 2014) and healthy young adults (Meinzer et al., 2013). These findings suggest that modulating the left DLPFC may influence interactions between the central executive network (CEN) and the default mode network (DMN). Both are large-scale networks with altered connectivity in aging and across the Alzheimer’s disease spectrum. Disruptions in DMN–CEN balance are consistently associated with poorer cognitive performance in older adults (Wang et al., 2007; Badhwar et al., 2017; Grady et al., 2016), making the left DLPFC a theoretically compelling target for intervention in MCI. rTMS applied to this region may help normalize dysregulated network dynamics and support higher-order cognitive processes vulnerable to early neurodegenerative change.

Beyond prefrontal stimulation, emerging work suggests that the lateral parietal cortex (LPC) may also be a promising site for intervention. In healthy young adults, five daily sessions of rTMS over this region significantly improved associative memory (Wang et al., 2014), supporting the idea that stimulating parietal regions involved in memory integration could complement prefrontal approaches. Considering that MCI affects distributed neural systems rather than isolated cortical regions, a broader stimulation strategy that includes both prefrontal and parietal cortex may ultimately yield more meaningful clinical effects.

### 4.3 Impact of TMS on cognition in individuals with AD

Compared with MCI, Alzheimer’s disease (AD) is characterized by greater network disruption and more heterogeneous responses to stimulation. Given the limited availability of effective treatments, TMS research has focused on symptom management and the pursuit of disease-modifying effects. Early studies indicate that rTMS can improve certain cognitive outcomes of AD, such as memory deficits and language function (Šimko et al., 2022; Wang et al., 2020). These effects are likely shaped by AD-related neurophysiological changes, including cortical hyperexcitability, reduced inhibitory function, and impaired LTP-like plasticity, which appear especially relevant to memory disfunction (Chou et al., 2022; Di Lorenzo et al., 2019)

Much like the variability seen in stimulation protocols for MCI, rTMS interventions in AD have employed diverse parameters, though most studies have stimulated the left DLPFC (Ahmed et al., 2012; Chu et al., 2021; Dong et al., 2018; Rutherford et al., 2015). Findings indicate that protocols stimulating multiple cortical sites may yield larger improvements in cognitive outcomes than those targeting a single region (Zhang et al., 2025). As in MCI, AD is characterized by disruptions of memory-related networks, notably the DMN and limbic networks (Krajcovicova et al., 2014; Gour et al., 2014). These networks link the molecular pathology of neurodegeneration with its clinical manifestations, suggesting that treatment effects may depend less on stimulation of an isolated cortical site than on the capacity of distributed networks to respond (Pini et al., 2018). Accordingly, TMS research in AD has shifted toward multisite stimulation aimed at the broader network pathophysiology of the disease (Pini et al., 2018).

This network-based perspective has prompted more spatially distributed stimulation approaches, such as the Neuro-AD™ system, which applies rTMS across multiple cortical regions alongside individualized cognitive training paradigms (Rabey et al., 2013; Rabey and Dobronevsky, 2016; Lee et al., 2016; Brem et al., 2020). Although this broadens the stimulation field, rTMS is still delivered to one area at a time rather than engaging interconnected regions simultaneously. As a result, it only partially addresses the network dysfunction central to AD, highlighting need for more integrative stimulation strategies capable of modulating distributed neural systems in a coordinated manner (Rabey et al., 2013; Rabey and Dobronevsky, 2016; Lee et al., 2016; Brem et al., 2020).

Stimulation frequency and disease severity both appear to shape therapeutic outcomes in AD. High-frequency stimulation over the left DLPFC and low-frequency stimulation over the right DLPFC have both been associated with significant memory improvement, although high-frequency protocols may produce stronger effects overall (Li et al., 2023; Turriziani et al., 2019). Whether combining rTMS with cognitive training enhances efficacy remains uncertain: some studies report greater improvement in cognitive outcomes with combined approaches (Wang et al., 2020), and others report no additional benefit beyond rTMS alone (Chu et al., 2021; Lin et al., 2019). This variability may partly reflect disease stage, as rTMS appears more effective in mild to moderate AD than in late-stage disease or MCI, where networks may be either too impaired or not yet sufficiently disrupted to benefit fully from stimulation (Mukli et al., 2023).

### 4.4 Clinical applications of rTMS in healthy aging, MCI and AD

The ability of rTMS to influence cortical activity beyond the duration of stimulation suggests potential utility across neurological and psychiatric disorders (Lefaucheur et al., 2020). It became a widely used clinical tool after FDA approval in 2008, for the treatment of medication-refractory major depressive disorder (McClintock et al., 2018). Since then, rTMS has been studied in Parkinson’s disease, dementia, neuropathy, and stroke (Lefaucheur et al., 2020; Lefaucheur et al., 2014)–and its clinical applications continue to expand. TMS has proven effectiveness (Level A evidence) for treating depression, motor stroke, and neuropathic pain, and it has received FDA approval for treating obsessive-compulsive disorder, smoking cessation, and migraines (Cohen et al., 2022); iTBS, a variant of rTMS, has also been approved for depression (Blumberger et al., 2018). Despite these advances, the application of TMS in cognitive impairment and dementia is still developing. Across this continuum, the same protocol is unlikely to perform uniformly. Differences in reserve, network integrity, and disease severity likely shape response to stimulation from healthy aging to MCI and AD. The applicability of rTMS across brain disorders may be attributed to three features: (i) existing connections between the cerebral cortex and other brain regions; (ii) its ability to modulate cortico-cortical and cortico-subcortical networks; and (iii) the flexibility of rTMS to tailor stimulation parameters to different targets (Barr et al., 2009; Blumberger et al., 2018; Caulfield and Brown, 2022; Chen et al., 1997b; Chou et al., 2020; Chu et al., 2021; Klomjai et al., 2015).

The clinical effects of rTMS on AD have been a subject of growing interest, particularly given the evidence of its neurochemical and neurobiological impacts as shown in previous parts of this review. However, TMS-induced neurochemical and neurobiological changes specific to AD remain incompletely understood. Moreover, while rTMS shows promise in clinical applications, its current standing in evidence-based guidelines is classified as level C evidence for the treatment of cognitive impairments in AD (Lefaucheur et al., 2020). This classification implies that, although there is some preliminary evidence suggesting rTMS could be beneficial for cognitive enhancement, the overall confidence in its effectiveness is relatively low (Lefaucheur et al., 2020). This is primarily due to the lack of standardization in rTMS protocols across studies, which complicates comparisons, and to substantial variability of results between individuals (Goldsworthy et al., 2021; Klomjai et al., 2015; Ziemann and Siebner, 2015).

Interindividual variability, already evident at the mechanistic and clinical levels, has increasingly pushed the field toward more personalized approaches, that take into account individual biological characteristics and specific biomarkers (Hampel et al., 2021; Menardi et al., 2022). Recent studies in depression treatment have shown benefits from individualized target selection, using various methods such as symptom-response mapping, targeting functionally derived brain parcels, or focusing on individual connectivity profiles of regions of interest (Moreno-Ortega et al., 2020; Cash et al., 2021). Applying these principles to AD and cognitive impairment is paramount. Efforts such as the Alzheimer Precision Medicine Initiative advance this goal by combining individual biochemical, functional, metabolic, morphological, and neuropsychological profiles into quantitative disease models (Hampel et al., 2019) and potentially refine treatment selection. While studies have confirmed the effects of rTMS in AD patients, individualized interventions based on anatomical MRI may not fully capture the neural rearrangements typical of the aging brain. Functional and structural brain organization varies significantly between individuals (Medaglia et al., 2020; Langs et al., 2016), and this is likely a key determinant of whether stimulation produces meaningful improvement in cognitive outcomes. This issue may be especially relevant in aging populations, where physiological brain changes further influence responsiveness to TMS (Cappon et al., 2022).

## 5 Safety, tolerability, and side effects of TMS

The most reported side effect of TMS is headache, which–along with neck and scalp pain–occurs in about 20–40% of patients undergoing both LF- and HF-rTMS treatments (Pagali et al., 2024). Though generally transient, these headaches can be a source of discomfort. More serious side effects, such as seizures, are very rare and occur in less than 1% of the time (Pagali et al., 2024). However, certain factors can increase this risk, including alcohol use, brain injury, sleep deprivation, family history of seizures, and others (Wassermann, 2000; Rossi et al., 2009). Although several case studies have described situations where these factors might contribute to an increased risk of seizures during TMS, a recent review (Lerner et al., 2019) of surveys from 174 TMS providers between 2012 and 2016 reported 24 seizures in 318,560 TMS sessions, highlighting the rarity of this adverse event in the context of TMS (Lerner et al., 2019).

Structural changes, histotoxicity, or tissue damage due to TMS are unlikely, although the possibility of unintended long-term brain changes cannot be entirely ruled out (Wassermann, 2000; Rossi et al., 2009). The clicking sound of the TMS device and the scalp sensation it produces can create multisensory effects and shift spatial attention (Duecker and Sack, 2015). It is also important to note that incorrect positioning of the TMS coil can result in overestimation of MT, placebo effects, or unintended impacts on behavioral, physiological, and cognitive processes (Duecker and Sack, 2015; Koehler et al., 2023).

To minimize the risk of side effects, adherence to safety guidelines for TMS is essential. Wassermann (Wassermann, 2000) and Chen and colleagues (Chen et al., 1997b) have provided recommendations on frequencies, current intensities, and trains of stimuli that are considered safe and effective. Additionally, using TMS parameters that involve short trains and longer intervals between trains reduces the risk of side effects (Rossini et al., 2015; Rossi et al., 2009). Given the vulnerability of older adults and patients with AD, it is particularly important to consider these safety guidelines and potential side effects when administering TMS. Unfortunately, due to data limitations, the long-term safety of neuromodulation is not frequently assessed, although available data indicate that rTMS is not associated with long-term side effects (Pagali et al., 2024).

## 6 Challenges and future directions

This section outlines key challenges that also define priority directions for future research, especially in the significant individual variability in response to stimulation, the lack of standardization in research protocols and devices, parameter optimization, heterogeneity in clinical trial designs, and the lack of clarity regarding the long-term efficacy and safety of TMS.

### 6.1 Standardization of TMS devices

Technological and methodological challenges in TMS research can impact the precision, effectiveness, and generalizability of findings. With the rise in TMS use and applications, the number of TMS devices commercialized has increased substantially in recent years (Cohen et al., 2022). To keep pace with this growth, regulatory controls have also been strengthened. International standards, such as those from the International Organization for Standardization (ISO), provide a technical basis for health, safety, and environmental regulations worldwide, and TMS devices are designed with these principles in mind (Lamph, 2012). Each country maintains its own regulations, but the European Union and the United States (through the FDA) have the most globally recognized standards (Food and Drug Administration, 2025; Lamph, 2012).

Most commercially available TMS devices comply with agency regulations (Cohen et al., 2022), despite the absence of specific standards dedicated to TMS device manufacturing. Several devices are designated for research use only and may not fully comply with manufacturing guidelines or existing certifications, such as FDA regulations and quality certificates. To illustrate, newly developed experimental TMS prototypes being commercialized offer greater control over pulse characteristics than clinical devices (Gutiérrez-Muto et al., 2023). Another example is the Theta-Burst protocol, which involves a notable trade-off between frequency and maximum stimulation intensity. This balance depends significantly on the TMS model used and can directly influence therapeutic effectiveness (Gutiérrez-Muto et al., 2020). Pulse shape remains a crucial determinant of TMS’s physiological impact. Recent devices enable greater control over stimulation waveforms, enabling more rectangular pulses and continuous parameters adjustment, like pulse width and the positive/negative phase amplitude ratio (Li et al., 2022; Gutiérrez-Muto et al., 2023). While these innovations provide increased flexibility, the lack of standardization and substantial differences in the technicalities of TMS (pulse duration and shape, output intensity, and coil designs) challenge the replication of study conditions across devices. This variability also complicates efforts toward individualized therapy and highlights the need for a comprehensive database to support cross-device comparisons and the development of unified standards.

### 6.2 Integration of TMS and neuroimaging

Accurately measuring the effects of TMS on brain activity and linking them to cognitive or clinical outcomes in healthy aging, MCI, and Alzheimer’s disease remains challenging. Although combining TMS with EEG, functional near-infrared spectroscopy (fNIRS), positron emission tomography (PET), or fMRI can provide critical insight into its neural effects, these multimodal approaches introduce substantial technical and methodological challenges. Nonetheless, such integration is necessary to refine stimulation protocols, capture both immediate and longer-lasting responses, and clarify how TMS influences behavior and cognition. One significant issue is the interference between TMS with imaging equipment, particularly in fMRI, where strong magnetic pulses introduce artifacts because the technique depends on stable field homogeneity.

Researchers can apply TMS offline, but this limits the ability to capture immediate neural responses. MR-compatible systems allow online experiments, although they require precise synchronization of TMS pulses with the fMRI acquisition sequence (Mizutani-Tiebel et al., 2022). In the inter-volume approach, stimulation is delivered during the brief pauses between successive volumes in an Echo Planar Imaging (EPI) sequence, and the timing is restricted by the repetition time (TR), which is commonly about 2 s (Bohning et al., 1998; Mizutani-Tiebel et al., 2022). The inter-slice method administers pulses between individual slice acquisition and supports stimulation frequencies up to 10 Hz, but it demands close coordination so that pulse timing does not interfere with the imaging process (Riddle et al., 2022). Maintaining this timing during fMRI adds substantial complexity. Additional challenges include limited coil maneuverability in the scanner, head motion that disrupts coil placement, and difficulties in designing sham conditions.

fNIRS is an alternative optical imaging method that is less prone to electromagnetic interference from TMS output. As a result, it can be used simultaneously with TMS without introducing the artifacts typically seen in fMRI (Mckendrick et al., 2015; Curtin et al., 2019). This makes it well-suited for tracking rapid hemodynamic changes that occur immediately after stimulation. However, fNIRS is restricted to superficial cortical regions and is sensitive to scalp and skull characteristics that can vary substantially in older adults (Okada and Delpy, 2003). These features can introduce noise into the measurements, although techniques such as short-separation channels help mitigate them and help measure superficial blood flow more accurately (Gagnon et al., 2011). In addition, fNIRS can also be seamlessly integrated with other forms of electrical brain stimulation, like tDCS (Mckendrick et al., 2015). Despite its limited depth resolution, fNIRS offers a cost-effective approach, as it is portable and allows use in scenarios where participants are free to move, such as in outdoor environments (Gateau et al., 2018; McKendrick et al., 2016).

EEG is another widely used modality that provides excellent temporal resolution and supports the measurement of TMS-evoked potentials (TEPs) and brain oscillations. Although EEG and TMS have been combined for more than two decades, methodological standardization remains limited, and differences in setup, recording, artifact correction, and analytic procedures make cross-study comparisons challenging (Ilmoniemi et al., 1997). Their integration also requires careful consideration of several factors, including the signal-to-noise ratio, TMS threshold determination, and management of responses caused by peripheral stimulation. Additional difficulties arise from TMS-induced somatosensory and auditory sensations that may affect EEG readings, as well as from the need to maintain stable coil placement during recording (Ilmoniemi and Kičić, 2010; van Doren et al., 2015a).

Beyond helping us understand how TMS affects brain function and behavior, integrating TMS with neuroimaging can guide real-time stimulation. Triggering TMS according to ongoing neural features, such as EEG phase or amplitude, may improve the precision and efficacy of stimulation (van Doren et al., 2015b; Hernandez-Pavon et al., 2023; Karabanov et al., 2016). Closed-loop systems continuously monitor neural activity, and the collected data is used to adjust TMS parameters in real time, tailoring the stimulation to the individual’s current state. Consider, for instance, in treating conditions like depression or epilepsy, closed-loop systems could detect specific brain activity patterns and deliver TMS pulses at the most opportune moments, maximizing the stimulation’s efficacy. This can also be seen in individuals with essential tremors, where closed-loop DBS has been found to be superior to open-loop DBS in reducing tremor intensity and concurrently lowering the stimulation-induced side effects (Sellers et al., 2024). In epilepsy, Liang et al. (2010) demonstrated that closed-loop DBS could detect epileptic seizures as they occur and respond with immediate suppression, offering a real-time therapeutic intervention (Liang et al., 2010). Despite the clinical value of closed-loop stimulation systems seen in other neurological conditions, their application to AD and cognitive impairment remains underexplored, probably because reliable real-time biomarkers of cognitive function have yet to be clearly established.

### 6.3 Inter-individual variability and implications for individualized stimulation

Response to stimulation with TMS varies substantially across individuals, and aging adds its own person-specific factors to consider when assessing the efficacy of rTMS for cognitive outcomes. Determining which patients are most likely to benefit based on their unique clinical and neurobiological characteristics presents a considerable challenge. Part of this inconsistency is compounded by the lack of standardized TMS protocols, as differences in stimulation frequency, intensity, duration, and cortical target further influence individual responses. Studies, including cluster analyses of responses to various non-invasive brain stimulation protocols, have highlighted a bimodal pattern of response (responders versus non-responders) (López-Alonso et al., 2014; van de ruit and Grey, 2019). Future work will therefore need to characterize which individuals are most responsive to stimulation and to identify the stimulation parameters most likely to yield meaningful cognitive benefits.

### 6.4 Future directions in TMS research for cognitive function

We would like to highlight some future directions for TMS research on cognitive impairment and AD:

#### 6.4.1 Identifying cognitive biomarkers and personalization of treatment

Identifying cognitive biomarkers and refining personalization strategies in for TMS cognitive decline in aging, MCI, and -AD is critical for enhancing both efficacy and precision. Advanced analytical methods, including neuroimaging techniques and machine learning algorithms, can be applied in both data-driven and theory-driven approaches (Liu et al., 2025). These methods help sift through large datasets to identify patterns and predictors that might not be apparent through traditional analysis. For instance, these approaches may be used to identify potential predictors of treatment response in depression (Nord, 2021). A similar strategy could be applied to cognitive impairment and AD, where neuroimaging data combined with machine learning could help identify specific brain patterns that predict response to stimulation (Espinosa et al., 2017; Boehm-Sturm et al., 2017).

#### 6.4.2 TMS protocols tailored to an individual’s specific neurovascular coupling profile

Evidence indicates that NVC is impaired in normal aging (Bowman et al., 2018; Lipecz et al., 2019; López-Otín et al., 2013; Mukli et al., 2023; Owens et al., 2023; Stefanova et al., 2013; Tarantini et al., 2017; Topcuoglu et al., 2009). Dysregulated NVC responses may contribute to the development of age-related MCI and dementia (Mukli et al., 2023; Owens et al., 2023; Zhu et al., 2022). Such alterations can occur before clinical symptoms emerge, as inadequate neurovascular regulation may precede neurodegeneration. These findings suggest that conventional approaches to evaluating cortical excitability may not be fully understood. In this context, understanding non-neural contributions to rTMS effects gains significance, especially regarding the potential impact on the brain’s vasculature (Cirillo et al., 2017). Aging-related NVC impairment also suggests why conventional measures of cortical excitability, including short intracortical inhibition and resting motor threshold, have not reliably predicted individual response to stimulation (López-Alonso et al., 2014). Integrating neuronal and vascular markers, informed by NVC assessments, represents a promising approach to personalizing TMS protocols. Tailoring stimulation to an individual’s NVC profile could enhance cognitive benefits while minimizing adverse effects, offering a biologically informed strategy for interventions targeting cognitive impairment in aging and AD.

Recent studies using advanced neurovascular imaging, such as fMRI, have demonstrated changes in the blood-oxygen level dependent (BOLD) response, which is an indicator of neurovascular function, before and after rTMS (Duarte et al., 2020; Min et al., 2016; Bestmann et al., 2005; Bestmann et al., 2004). Further investigations suggest that rTMS can modulate CBF and support blood-brain barrier integrity (Zong et al., 2020; Zhang S. et al., 2022). Functional near-infrared spectroscopy (fNIRS) in humans indicates that NVC involves an interplay of vascular and neural components, with TMS-induced NRS changes capturing both local vascular effects and neuronal activity (Näsi et al., 2011; Paus et al., 1998). However, evidence regarding the role of vascular and non-neuronal factors in shaping TMS outcomes, especially in age-related cognitive decline, MCI, and AD, remains limited.

By evaluating NVC and its correlation with cognitive performance, researchers can establish individualized neurobiological profiles that capture both neuronal and vascular characteristics (Näsi et al., 2011; Paus et al., 1998; Zhu et al., 2022). These profiles enhance understanding of patient-specific response to stimulation and provide a foundation for developing more personalized and effective interventions (Cirillo et al., 2017; López-Alonso et al., 2014). Tailoring TMS protocols to an individual’s specific NVC profile could potentially enhance cognitive benefits while reducing adverse effects (Lipecz et al., 2019; Mukli et al., 2023). To improve treatment of cognitive impairment in AD, future interventions should move beyond anatomical targeting and incorporate functional and structural connectivity profiles. This would allow a more precise characterization of brain organization and NVC responses (Bestmann et al., 2004; Duarte et al., 2020; Min et al., 2016). This approach not only allows insights into CBF dynamics in response to neuronal activity but also positions NVC as a potential biomarker in TMS trials (Bowman et al., 2018; Owens et al., 2023). Integrating NVC-informed profiling could provide a holistic understanding of brain function and its implications for tailored therapeutic strategies in cognitive impairment and AD.

#### 6.4.3 Integration with neuroimaging

Building on the methodological considerations discussed above, the integration of TMS with neuroimaging techniques can greatly enhance our understanding of the neural mechanisms underlying TMS’s effects on cognitive impairment and AD. For instance, neuroimaging biomarkers of brain structure and function guide treatment selection in major depressive disorder (Fonseka et al., 2018). In this context, fNIRS offers distinct advantages over traditional methods like fMRI. The option for a three-way combination with fNIRS/EEG/TMS can provide comprehensive insights into both the hemodynamic (via fNIRS) and electrical (via EEG) responses of the brain to TMS, offering a more holistic understanding of the brain’s functional changes (Curtin et al., 2019). This could lead to more effective, personalized TMS protocols, especially in conditions where NVC may play a crucial role, such as in aging, MCI, and AD.

## 7 Discussion

TMS has emerged as a compelling non-pharmacological intervention for modulating cognitive outcomes, especially in aging and neurodegenerative diseases such as AD, yet the evidence synthesized in this review reveals a field marked by both encouraging progress and considerable uncertainty. Across studies, rTMS appears capable of engaging neuroplastic mechanisms that influence memory, attention, and executive function (Abellaneda-Perez et al., 2022; Barr et al., 2009; Cheng et al., 2018; Chou et al., 2020; Chu et al., 2021; Dong et al., 2018; Gaudeau-Bosma et al., 2013; Kim et al., 2012; Lin et al., 2019; Patel et al., 2020; Pearce et al., 2014; Šimko et al., 2022; Wang et al., 2021; Wang et al., 2020; Zhang et al., 2021). However, the strength and durability of these effects depend on multiple biological and methodological factors.

The literature suggests that stimulation of the DLPFC can modulate processes related to encoding and retrieval (Patel et al., 2020), and findings in older adults indicate that age-related shifts in hemispheric organization may shape the response to stimulation (Rossi et al., 2004; Manenti et al., 2011). Studies reporting disrupted retrieval following both left and right DLPFC stimulation in older adults, together with preserved left-hemisphere dominance for encoding, support broader frameworks such as the HAROLD model (Cabeza, 2002; Cabeza et al., 2002; Rossi et al., 2004). In this context, network-level effects of rTMS in healthy aging are more plausibly interpreted as compensatory than strictly restorative. This interpretation is supported by findings showing increased prefrontal activation following treatment in older adults with subjective memory complaints and lower baseline retrieval performance (Solé-Padullés et al., 2006; Cui et al., 2020).

Despite these compelling observations, the literature on healthy aging presents mixed evidence regarding the effectiveness of rTMS. While several studies describe improvements in memory, attention, or processing speed, meta-analytic data paint a more restrained picture, with small or negligible effects across most cognitive domains and modest improvements primarily in executive function (Patel et al., 2020). This discrepancy likely reflects the heterogeneity of cognitive aging, and methodological variation across studies. Older adults differ widely in cognitive reserve, neural resilience, and vulnerability to age-related change, all of which may shape response to stimulation (Cabeza et al., 2002; Cappon et al., 2022; Langs et al., 2016; Medaglia et al., 2020). This pattern is consistent with a state-dependent interpretation, in which the response to stimulation vary accordingly to the baseline functional organization of the aging brain (Silvanto et al., 2008). Some individuals perform comparably to younger adults on memory tasks, which may reduce the measurable effects of stimulation and contribute to the muted effect sizes reported in aggregated analyses (Cabeza et al., 2002). Therefore, stratified approaches that account for individual differences are needed, rather than assuming a uniform response to stimulation in healthy older populations.

In MCI, evidence for rTMS to affect general cognitive outcomes or specific cognitive domains remains mixed. Many available studies rely on small cohorts and short-term follow-up, making it difficult to determine the durability of rTMS-related cognitive effects. Several clinical trials report improvements in general cognition or memory that surpass sham stimulation (Šimko et al., 2022; Teselink et al., 2021; Chu et al., 2021; Wang et al., 2021; Dong et al., 2018; Wang et al., 2020; Lin et al., 2019; Zhang et al., 2021; Cheng et al., 2018; Chou et al., 2020); others show more selective domain-specific effects or no significant benefits (Chu et al., 2021). This variation likely reflects both differences in how cognitive domains are operationalized across studies, where constructs such as “memory,” “executive function,” or “global cognition” are assessed through different tests and endpoints, and broader methodological heterogeneity, including stimulation parameters, study duration, cognitive baselines, sample sizes, and disease characteristics (Beishon et al., 2021; Chu et al., 2021; Lin et al., 2019; Mukli et al., 2023; Wang et al., 2020).

Most studies focus on the left DLPFC, based on evidence from psychiatry and aging research implicating this region in regulating interactions between the central executive network and the default mode network (DMN) (Liston et al., 2014; Solé-Padullés et al., 2006; Wang et al., 2021). Emerging network-mapping work in MCI suggests that prefrontal stimulation may primarily engage the salience network, whereas parietal stimulation may more directly engage posterior default network regions while also modulating frontoparietal systems (Taylor et al., 2025). Disruptions in these networks are strongly associated with cognitive decline in aging (Wang et al., 2007; Badhwar et al., 2017; Grady et al., 2016). This network specificity also supports a state-dependent interpretation of TMS effects in MCI, as the impact of stimulation is likely to vary according to the baseline functional state of the network being engaged (Silvanto et al., 2008). Modulating the left DLPFC remains a common approach, yet MCI affects distributed neural systems, and broader stimulation strategies, including lateral parietal cortex (LPC) involvement, may yield more meaningful results (Wang et al., 2014).

For Alzheimer’s disease (AD), the rTMS literature points to potential positive effects on cognitive outcomes, including improvements in memory and language, although results remain variable and often depend on stimulation frequency, cortical targets, and intervention duration (Šimko et al., 2022; Wang et al., 2020). High-frequency stimulation of the left DLPFC is the most common approach (Ahmed et al., 2012; Chu et al., 2021; Dong et al., 2018; Rutherford et al., 2015), yet protocols involving multiple cortical regions may produce larger effects and better engage the distributed networks disrupted in AD (Zhang et al., 2025). This shift toward network-based interventions reflects growing recognition that AD pathology affects broad neural systems rather than isolated cortical sites. Accordingly, network-level effects in AD are best viewed as only partially restorative and strongly-state dependent, as response to stimulation likely depends on residual network integrity and capacity for reorganization (Bashir et al., 2022; Mukli et al., 2023; Silvanto et al., 2008). Findings showing rTMS-related modulation of dopaminergic signaling and NMDA receptor expression (Choung et al., 2021; D’Amelio et al., 2018; Malik et al., 2018; Pan et al., 2019; Tan et al., 2013; Zhang Y. et al., 2016) add a mechanistic dimension to these clinical observations, though it remains uncertain how reliably these neurobiological changes translate into symptom improvement in humans.

The Neuro-AD™ protocol illustrates an applied attempt to bridge this mechanistic and network-level gap, yet its sequential stimulation approach does not fully capture the coordinated, simultaneous modulation that network dysfunction in AD may require (Brem et al., 2020; Lee et al., 2016; Rabey and Dobronevsky, 2016; Rabey et al., 2013). Interpretation of these findings is further limited by heterogeneity in disease stage, stimulation parameters, outcome measures, including inconsistency in how cognitive domains are defined and assessed across studies, as well as the use of small samples and short follow-up (Ahmed et al., 2011; Alcalá-Lozano et al., 2018; Dengler et al., 2024; Fazio et al., 2025; Hu et al., 2022; Lv et al., 2023; Yao et al., 2022). In this context, statistically significant changes in cognitive tests should be interpreted with caution and do not necessarily reflect clinically meaningful changes in a progressive disorder such as AD. These limitations suggest that, while findings are encouraging, the therapeutic role of rTMS in AD remains to be fully established. Further innovation in protocol design is needed to match the complexity of AD-related changes in brain organization.

A recurring theme across the evidence is substantial interindividual variability in response to rTMS. Rather than attributing this solely to methodological inconsistency, these divergent outcomes highlight the critical role of state-dependency and disease severity. The efficacy of rTMS likely relies on a “functional reserve,” both neural and vascular, that varies across the cognitive continuum. Evidence suggests a non-linear relationship with severity: rTMS appears most effective in individuals with mild to moderate AD, where plasticity is impaired but the structural substrate remains sufficiently intact to support reorganization (Mukli et al., 2023). In late-stage disease, profound atrophy may render the cortex unresponsive (a “ceiling effect”), whereas in MCI, the subtle nature of network disruptions may require more precise, connectivity-informed targeting to elicit measurable gains. Therefore, stratification based on neural and vascular reserve is essential to identify “responders” capable of supporting the metabolic demands of induced plasticity. These differences raise awareness of patient selection criteria and the possibility that personalized approaches informed by biological, cognitive, or imaging markers may be necessary to optimize treatment effects.

Neurovascular coupling (NVC) represents an emerging biomarker that may clarify individual variability in the response to stimulation by capturing the dynamic relationship between neuronal activity and cerebral blood flow. Studies show that NVC influences cognitive performance and may change in aging, MCI, and AD (Näsi et al., 2011; Owens et al., 2022; Paus et al., 1998; Zhu et al., 2022). Integrating NVC measures with TMS protocols represents a promising direction to tailor interventions to the neurobiological profile of each individual and improve both precision and efficacy (Cirillo et al., 2017; Lipecz et al., 2019; López-Alonso et al., 2014; Mukli et al., 2023). Moreover, combining TMS with neuroimaging tools such as fNIRS and EEG offers a powerful framework for capturing concurrent hemodynamic and electrical responses, deepening understanding of how TMS influences neural systems in real time (Curtin et al., 2019). Multimodal approaches combining TMS with neuroimaging and electrophysiology may represent a key direction for guiding targeted therapeutic decisions and addressing mechanistic questions that have historically limited the field.

Taken together, the literature supports cautious optimism regarding the role of TMS in cognitive aging and neurodegeneration. While the mechanistic and clinical findings are encouraging, significant challenges remain. Methodological heterogeneity, small sample sizes, limited long-term follow-up, and variability in stimulation targets reduce confidence in effect estimates. Differences in study design and in the way cognitive domains are measured further complicate cross-study interpretation. Controversies persist regarding optimal parameters, the value of multisite versus single-site approaches, and whether rTMS-related improvements reflect meaningful changes in cognitive outcomes or compensatory processes that may wane over time. These uncertainties highlight the importance of rigorous future studies with standardized protocols, biomarkers to stratify responders, and designs capable of probing both network-level and vascular contributions to treatment outcomes.

TMS holds substantial potential as a therapeutic modality to support cognitive health across the spectrum from healthy aging to Alzheimer’s disease. Its ability to modulate neural activity, engage plasticity mechanisms, and influence network dynamics offers a compelling therapeutic pathway, yet its full clinical potential will depend on continued fine-tuning of stimulation strategies, exploration of multisite protocols, and deeper mechanistic insight. Future work should focus on integrating multimodal biomarkers, including neurophysiological and neurovascular measures, with stimulation protocols to better define responders and guide individualized interventions. In parallel, combining neuromodulation with cognitive or behavioral interventions may further enhance treatment effects and improve clinical relevance.
